# Epidemiology and Outcomes of Sporotrichosis: A Descriptive Real‐World Analysis From a Global Cohort

**DOI:** 10.1111/myc.70152

**Published:** 2026-02-24

**Authors:** Samantha Schapiro, Nicholas Pulciano, Julian Galindo‐Ramirez, Nicholas Rau, Jose Tuells, Nelson Iván Agudelo Higuita, George R. Thompson, Daniel B. Chastain, Andrés F. Henao‐Martínez

**Affiliations:** ^1^ Saint Joseph Hospital Internal Medicine Residency Denver Colorado USA; ^2^ Rocky Vista University College of Osteopathic Medicine Parker Colorado USA; ^3^ Internal Medicine Residency Program University of Texas Rio Grande Valley Texas USA; ^4^ Division of Infectious Diseases, Department of Medicine University of Colorado Denver Aurora Colorado USA; ^5^ School of Health Sciences Universidad Cardenal Herrera‐CEU, CEU Universities Valencia Spain; ^6^ Division of Infectious Diseases, Department of Medicine The University of Oklahoma Health Campus Oklahoma City Oklahoma USA; ^7^ Instituto de Enfermedades Infecciosas y Parasitología Antonio Vidal Tegucigalpa Honduras; ^8^ University of California‐Davis, Medical Center Sacramento California USA; ^9^ Department of Clinical and Administrative Pharmacy UGA College of Pharmacy Augusta Georgia USA

**Keywords:** antifungal agents, HIV infections, mortality, mycoses, sporotrichosis, United States

## Abstract

**Background:**

Sporotrichosis is a dimorphic fungal infection of increasing global relevance. Although usually cutaneous or lymphocutaneous, disseminated disease occurs in immunocompromised hosts. Large‐scale data on its epidemiology, treatment and outcomes remain limited.

**Objectives:**

To characterise the clinical features, comorbidities, antifungal prescribing patterns and one‐year mortality associated with sporotrichosis, with emphasis on patients with HIV and other forms of immunosuppression.

**Patients/Methods:**

We performed a retrospective global cohort study using the TriNetX Research Network. Adults (≥ 18 years) diagnosed with sporotrichosis were identified using ICD‐9/10 codes (1995–2024). Demographics, comorbidities, laboratory parameters and antifungal prescriptions were analysed. The primary outcome was all‐cause mortality at 1 year. Secondary outcomes included hospitalisation and admission to the intensive care unit (ICU).

**Results:**

Among 2124 adults, the mean age was 52 years, and 56.5% were female. Nearly half of the cases originated internationally, with the southeastern United States accounting for the majority of domestic cases. Neoplasms (25%) and diabetes (11%) were the most common comorbidities. Lymphocutaneous disease was uncommon (9%), and disseminated infection occurred in 1% of cases. One‐year mortality was 17%, with a higher risk among older adults and those with neoplasms, lymphocutaneous or disseminated infection or hyperferritinemia. Seventeen patients (0.8%) had HIV and were more likely to have pulmonary or disseminated disease. Itraconazole was the most commonly prescribed antifungal (52%), while the use of amphotericin B remained low (< 2%).

**Conclusions:**

Sporotrichosis causes substantial global mortality. Outcomes appear driven by host factors and gaps in guideline‐based management. Earlier recognition, optimised antifungal therapy, and use of inflammatory markers to guide risk stratification may improve outcomes and inform prevention strategies.

## 1 Introduction

Sporotrichosis is a subacute or chronic fungal infection caused by *Sporothrix* species, a group of dimorphic fungi ubiquitous in soil, plants, decaying vegetation and sphagnum moss [1, 2, 3]. Human infection typically follows traumatic inoculation, although inhalation of conidia or zoonotic transmission, particularly from infected cats or armadillos, has been increasingly recognised [2, 4]. Clinical manifestations range from cutaneous and lymphocutaneous disease to pulmonary or disseminated infection, with cutaneous forms accounting for most cases worldwide [1, 5]. Definitive diagnosis requires fungal culture demonstrating thermal dimorphism.

Although sporotrichosis has historically been associated with gardeners and agricultural workers, disseminated disease primarily affects individuals who are immunocompromised. People at highest risk include those with human immunodeficiency virus (HIV), poorly controlled diabetes mellitus, active malignancy, chronic glucocorticoid use, treatment with tumour necrosis factor (TNF)‐α inhibitors, alcohol use disorder or recipients of solid organ transplants [2, 6]. These conditions impair cell‐mediated immunity, increasing susceptibility to invasive disease [3]. Among people with HIV, disseminated disease occurs in up to 70%–89% of cases [3, 7], typically in those with profound immunosuppression (CD4 < 150 cells/μL) [3, 7, 8]. Mortality is estimated to be over 40‐fold greater than in HIV‐negative individuals [9]. Sporotrichosis in this population is frequently atypical and severe, often involving the central nervous system, lungs, mucosae, eyes and osteoarticular system, and is commonly associated with poor clinical outcomes [7, 8, 10].

Despite its global distribution, sporotrichosis remains underrecognised, in part because it is not a reportable disease in most countries [11]. Consequently, large‐scale epidemiologic and outcome data are scarce, particularly beyond the endemic regions of South America. This study aimed to characterise the clinical features, comorbidities and one‐year mortality associated with sporotrichosis using a global real‐world database.

## 2 Methods

### 2.1 Data Source

We conducted a retrospective cohort study using the TriNetX Global Health Research Network (https://trinetx.com/), a federated database aggregating de‐identified electronic health record (EHR) data from over 250 healthcare organisations (HCOs) across North America, Europe, Asia, Australia and South America. Participating institutions include large academic medical centres and integrated health systems, collectively representing more than 250 million patients.

TriNetX standardises data from participating HCOs into a standard data model and applies rigorous quality control procedures to ensure completeness and consistency. Data contributions include demographics, diagnoses, procedures, medications and laboratory results. Most institutions refresh data every 1–4 weeks, with an average latency of approximately 1 month.

TriNetX complies with the U.S. Health Insurance Portability and Accountability Act (HIPAA) and equivalent international data privacy regulations, and is certified under the ISO 27001:2013 standard for information security. All data are de‐identified under §164.514(a)–(b) of the HIPAA Privacy Rule, with expert determination of non‐identifiability. Because investigators cannot access identifiable data, this study was considered exempt from institutional review board oversight (Colorado Multiple Institutional Review Board [COMIRB#24‐1919], University of Colorado Denver).

### 2.2 Study Design and Population

Adult patients (≥ 18 years) diagnosed with sporotrichosis between 1995 and 2024 were identified using International Classification of Diseases (ICD)‐10‐CM code B42 or ICD‐9‐CM code 117.1 (Table S1). The earliest recorded diagnosis was designated as the index date.

To assess the accuracy of the diagnostic codes, we conducted an internal validation on 31 randomly selected institutional cases, comparing ICD‐coded diagnoses with microbiologic or histopathologic confirmation. The positive predictive value was 58% for culture‐ or biopsy‐confirmed cases and 90% when clinically suspected cases were included. The European Organization for Research and Treatment of Cancer and the Mycoses Study Group Education and Research Consortium (EORTC/MSGERC) criteria for proven sporotrichosis (histopathologic or culture evidence from an affected site or a positive blood culture) are not uniformly available within TriNetX and therefore could not be applied [12].

### 2.3 Variables and Data Collection

Demographic variables (age, sex, race, ethnicity, marital status and geographic region) were recorded at the index encounter. Comorbidities were identified using ICD‐10‐CM codes documented before the index date (Table S2). Laboratory data were extracted using Logical Observation Identifiers Names and Codes (LOINC) from 6 days before to 5 days after diagnosis (Table S3). Antifungal therapy was identified using RxNorm codes and assessed from 30 days before diagnosis to 90 days after diagnosis (Table S4). Procedures and clinical outcomes, including hospitalisation and intensive care unit (ICU) admission, were identified using Current Procedural Terminology (CPT), ICD‐10‐CM and Health Level Seven (HL7) terminologies, and evaluated from 15 days before to 60 days after diagnosis (Table S5).

### 2.4 Outcomes

The primary outcome was one‐year all‐cause mortality following the index diagnosis, ascertained from linked death indices, institutional EHR documentation and discharge disposition data recorded within the TriNetX system. Patients with missing information on mortality, demographics or comorbidities were excluded. Secondary outcomes included hospitalisation and ICU admission within 15 days before and 60 days after diagnosis.

For subgroup analyses, patients with HIV were identified by ICD‐10‐CM code B20. Available CD4 counts were captured from 6 days before to up to 5 days following the index diagnosis.

### 2.5 Statistical Analysis

Descriptive statistics were used to summarise demographic, clinical and laboratory variables. Continuous variables were reported as means with standard deviations (SD) and compared using *t*‐tests or Mann–Whitney *U* tests, as appropriate. Categorical variables were summarised as counts and percentages and compared using chi‐square or Fisher's exact tests. Statistical significance was defined as a two‐tailed *p* value < 0.05.

Comparative analyses were performed between survivors and non‐survivors at 1 year, US cases vs. international cases, and between individuals with and without HIV. We then performed a multivariable Cox regression analysis for 1‐year mortality. Geographic distributions of US cases were visualised using Datawrapper software (https://www.datawrapper.de/). All analyses were conducted in STATA 19.5 version (StataCorp LLC, College Station, TX) with individualized data.

### 2.6 Data Access

The corresponding author had full access to the study data and was ultimately responsible for deciding to submit the manuscript for publication. The individualised datasets generated and analysed in the current study are available from the corresponding author upon request.

### 2.7 Ethics Statement

TriNetX provides only de‐identified data, determined by an independent expert under 45 CFR §164.514(b)(1). Geographic reporting is limited to regional levels to minimise the risk of re‐identification. Research using the platform does not involve human subjects as defined by federal regulations; this project complied with HIPAA and was approved by COMIRB.

## 3 Results

### 3.1 Cohort Characteristics

**FIGURE 1:**
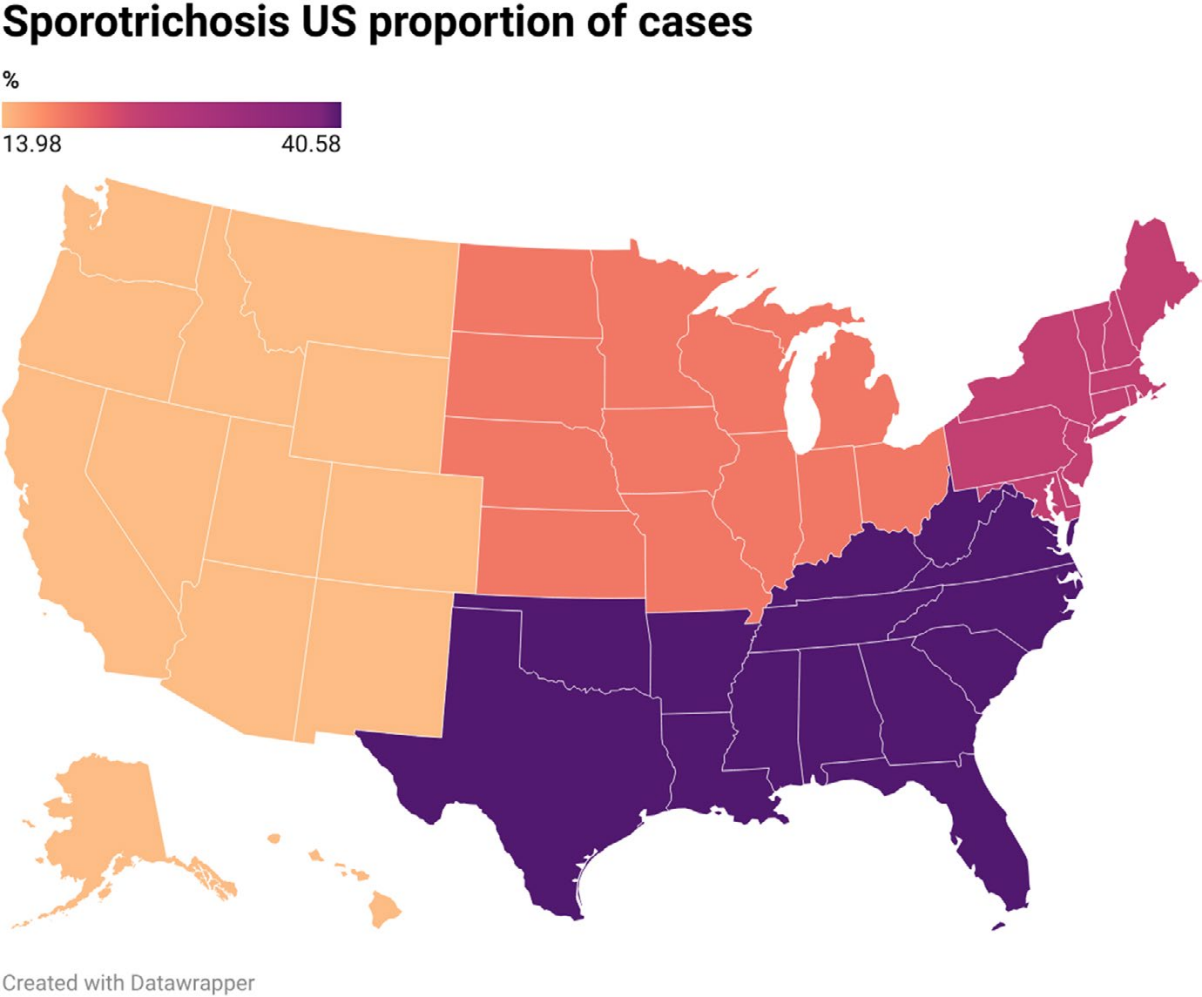
The proportion of Sporotrichosis cases in the United States.

A total of 2124 adults with sporotrichosis were identified. The mean age was 52 ± 19 years, and 56.5% were female (Table 1). Nearly half of the cases originated from international sites (49.3%), while within the United States, the Southeast region accounted for 21% of cases (Figure 1). The most common comorbidities were neoplasms (25.2%), type 2 diabetes mellitus (11.4%) and chronic obstructive pulmonary disease (COPD) (7.9%).

**TABLE 1:** Baseline characteristics and 1‐year outcomes among patients with sporotrichosis, by survival status.

Clinical features	Survivors (*N* = 1763)	Non‐Survivors (*N* = 361)	Total (*N* = 2124)	*p*
Demographics				
Age (years), mean (SD)	51.5 (18.4)	54.4 (21.6)	52.0 (19.0)	0.009
Sex				
Female	1009 (57.2%)	191 (52.9%)	1200 (56.5%)	0.304
Male	693 (39.3%)	155 (42.9%)	848 (39.9%)	
Unknown	61 (3.5%)	15 (4.2%)	76 (3.6%)	
Race				
White	719 (40.8%)	110 (30.5%)	829 (39.0%)	0.002
Black	56 (3.2%)	7 (1.9%)	63 (3.0%)	
Asian	20 (1.1%)	1 (0.3%)	21 (1.0%)	
American Indian	2 (0.1%)	0 (0.0%)	2 (0.1%)	
Native Hawaiian	1 (0.1%)	0 (0.0%)	1 (0.0%)	
Another race	73 (4.1%)	17 (4.7%)	90 (4.2%)	
Unknown	892 (50.6%)	226 (62.6%)	1118 (52.6%)	
Ethnicity				
Not Hispanic	634 (36.0%)	85 (23.5%)	719 (33.9%)	< 0.001
Hispanic	76 (4.3%)	11 (3.0%)	87 (4.1%)	
Unknown	1053 (59.7%)	265 (73.4%)	1318 (62.1%)	
Marital Status				
Single	780 (44.2%)	209 (57.9%)	989 (46.6%)	< 0.001
Married	444 (25.2%)	73 (20.2%)	517 (24.3%)	
Region				
International	811 (46.0%)	236 (65.4%)	1047 (49.3%)	< 0.001
Southeast	391 (22.2%)	54 (15.0%)	445 (21.0%)	
Northeast	231 (13.1%)	27 (7.5%)	258 (12.1%)	
Midwest	185 (10.5%)	14 (3.9%)	199 (9.4%)	
West	125 (7.1%)	26 (7.2%)	151 (7.1%)	
Comorbidities				
Neoplasm	410 (23.3%)	125 (34.6%)	535 (25.2%)	< 0.001
Type 2 diabetes	207 (11.7%)	36 (10.0%)	243 (11.4%)	0.336
COPD	150 (8.5%)	18 (5.0%)	168 (7.9%)	0.024
Chronic kidney disease	145 (8.2%)	21 (5.8%)	166 (7.8%)	0.121
Heart failure	109 (6.2%)	18 (5.0%)	127 (6.0%)	0.382
Inflammatory bowel disease	99 (5.6%)	17 (4.7%)	116 (5.5%)	0.49
Systemic connective tissue disease	51 (2.9%)	3 (0.8%)	54 (2.5%)	0.023
Transplant	44 (2.5%)	7 (1.9%)	51 (2.4%)	0.529
Liver disease	42 (2.4%)	4 (1.1%)	46 (2.2%)	0.13
Pulmonary fibrosis	24 (1.4%)	8 (2.2%)	32 (1.5%)	0.225
Aplastic anaemia	18 (1.0%)	10 (2.8%)	28 (1.3%)	0.008
HIV	14 (0.8%)	3 (0.8%)	17 (0.8%)	0.943
Antifungal therapy				
Itraconazole	416 (52.9%)	67 (45.3%)	483 (51.7%)	0.087
Fluconazole	59 (7.5%)	11 (7.4%)	70 (7.5%)	0.975
Posaconazole	13 (1.7%)	4 (2.7%)	17 (1.8%)	0.381
Voriconazole	8 (1.0%)	4 (2.7%)	12 (1.3%)	0.095
Isavuconazole	1 (0.1%)	3 (2.0%)	4 (0.4%)	0.001
Amphotericin B	15 (1.9%)	3 (2.0%)	18 (1.9%)	0.923
Echinocandin	5 (0.6%)	2 (1.4%)	7 (0.7%)	0.355
Laboratory values, mean (SD)				
WBC (×10^3^ cells/μL)	7.7 (3.0)	10.8 (7.2)	8.4 (4.5)	< 0.001
Haemoglobin (g/dL)	13.2 (1.8)	12.7 (2.4)	13.1 (1.9)	0.053
Haematocrit (%)	39.8 (5.0)	37.7 (9.2)	39.4 (6.1)	0.047
Platelets (×10^3^ cells/μL)	247.7 (88.6)	256.3 (113.0)	249.3 (93.4)	0.596
ALT (U/L)	29.9 (35.8)	34.3 (33.6)	30.7 (35.4)	0.487
AST (U/L)	28.1 (27.4)	30.7 (24.1)	28.6 (26.7)	0.531
Creatinine (mg/dL)	1.0 (0.7)	1.2 (1.1)	1.0 (0.8)	0.074
CRP (mg/L)	29.7 (58.4)	51.7 (68.9)	34.1 (60.8)	0.196
ESR (mm/H)	24.1 (26.7)	23.4 (16.8)	24.0 (25.2)	0.941
Ferritin (ng/mL)	209.1 (251.7)	3453.4 (4588.3)	904.3 (2279.5)	0.022
LDH (U/L)	209.1 (102.9)	344.0 (579.4)	234.4 (237.0)	0.393
Procalcitonin (ng/mL)	0.3 (0.4)	1.1 (1.0)	0.7 (0.8)	0.137
Outcomes				
Hospitalisation	12 (0.8%)	2 (0.6%)	14 (0.8%)	0.71
ICU admission	14 (0.9%)	2 (0.6%)	16 (0.9%)	0.559

Abbreviations: ALT, alanine aminotransferase; AST, aspartate aminotransferase; COPD, chronic obstructive pulmonary disease; CRP, C‐reactive protein; ESR, erythrocyte sedimentation rate; HIV, human immunodeficiency virus; ICU, intensive care unit; LDH, lactate dehydrogenase; WBC, white blood cell count.

Lymphocutaneous sporotrichosis was the most common identifiable clinical form (9.4%), followed by pulmonary (2.8%), disseminated (1.1%), and osteoarticular disease (1.0%). Itraconazole was the most commonly prescribed antifungal (51.7%), followed by fluconazole (7.5%), posaconazole (1.8%), voriconazole (1.3%) and amphotericin B (1.9%), which were used less frequently. The overall one‐year mortality rate was 17%, and under 1% of patients required hospitalisation or ICU admission. Compared to international sporotrichosis cases, cases in the United States predominantly affected younger women with malignancy‐related immunosuppression (particularly solid‐organ or hematologic malignancy, 29% vs. 22%, hematologic malignancy 5.7% vs. 3.5%; and lymphatic disease 11% vs. 8%) and were associated with a nearly two‐fold higher 1‐year mortality than cases reported from international centres (22.5% vs. 11.6%; *p* < 0.001).

### 3.2 Predictors of Mortality

Non‐survivors were older than survivors (54.5 vs. 51.5 years, *p* = 0.009) and had higher rates of neoplastic disease (34.6% vs. 23.3%, *p* < 0.001) and aplastic anaemia (2.8% vs. 1.0%, *p* = 0.008). Lymphocutaneous (12.5% vs. 8.8%, *p* = 0.029) and disseminated (2.5% vs. 0.8%, *p* = 0.004) disease were significantly more common among non‐survivors. Laboratory values in non‐survivors demonstrated higher white blood cell counts (10.8 vs. 7.7 × 10^3^ cells/μL, *p* < 0.001), lower haematocrit (37.7% vs. 39.8%, *p* = 0.047), and markedly elevated serum ferritin levels (3453 vs. 209 ng/mL, *p* = 0.022).

Antifungal prescribing patterns were similar between groups. Itraconazole was the most common therapy among both survivors (52.9%) and non‐survivors (45.3%), while isavuconazole use was more frequent among non‐survivors (2.0% vs. 0.1%, *p* = 0.001). Hospitalisation and ICU admission were rare (< 1%) and did not differ by survival status. In the multivariable Cox regression analysis, age, gender, ethnicity, region, underlying neoplasm and HIV infection were not independently associated with mortality in the adjusted model (Table S6). A lower haematocrit at diagnosis was significantly associated with increased 1‐year mortality (HR 0.94 per 1% increase, 95% CI 0.89–0.99; *p* = 0.012). Disseminated disease showed a trend toward higher mortality (HR 3.72, 95% CI 0.82–16.96; *p* = 0.090).

### 3.3 Sporotrichosis in People With HIV

Seventeen patients (0.8%) were living with HIV. They were predominantly male (82.4% vs. 39.6%, *p* = 0.002) and more commonly located in the Southern (41.2% vs. 20.8%) and Western (29.4% vs. 6.9%) United States (*p* < 0.001 overall) (Table 2). Compared with patients without HIV, those with HIV had higher rates of diabetes mellitus (35.3% vs. 11.2%, *p* = 0.002), COPD (23.5% vs. 7.8%, *p* = 0.017), chronic kidney disease (29.4% vs. 7.6%, *p* < 0.001), liver disease (17.6% vs. 2.0%, *p* < 0.001) and inflammatory bowel disease (17.6% vs. 5.4%, *p* = 0.026). The mean CD4 count among people with HIV was 65.5 cells/μL.

**TABLE 2:** Clinical characteristics and outcomes of patients with sporotrichosis, by HIV status.

Clinical features	HIV‐negative (*N* = 2107)	HIV‐positive (*N* = 17)	Total (*N* = 2124)	*p*
Demographics				
Age (years), mean (SD)	52.0 (19.1)	56.5 (14.4)	52.0 (19.0)	0.325
Sex				
Women	1197 (56.8%)	3 (17.6%)	1200 (56.5%)	0.002
Men	834 (39.6%)	14 (82.4%)	848 (39.9%)	
Unknown	76 (3.6%)	0 (0.0%)	76 (3.6%)	
Race				
White	820 (38.9%)	9 (52.9%)	829 (39.0%)	0.054
Other race	89 (4.2%)	1 (5.9%)	90 (4.2%)	
Black	61 (2.9%)	2 (11.8%)	63 (3.0%)	
Asian	20 (0.9%)	1 (5.9%)	21 (1.0%)	
American Indian	2 (0.1%)	0 (0.0%)	2 (0.1%)	
Native Hawaiian	1 (0.0%)	0 (0.0%)	1 (0.0%)	
Unknown	1114 (52.9%)	4 (23.5%)	1118 (52.6%)	
Ethnicity				
Not Hispanic	711 (33.7%)	8 (47.1%)	719 (33.9%)	0.005
Hispanic	84 (4.0%)	3 (17.6%)	87 (4.1%)	
Unknown	1312 (62.3%)	6 (35.3%)	1318 (62.1%)	
Marital status				
Single	985 (46.7%)	4 (23.5%)	989 (46.6%)	0.024
Married	514 (24.4%)	3 (17.6%)	517 (24.3%)	
Unknown	608 (28.9%)	10 (58.8%)	618 (29.1%)	
Region				
International	1046 (49.6%)	1 (5.9%)	1047 (49.3%)	< 0.001
Southeast	438 (20.8%)	7 (41.2%)	445 (21.0%)	
Northeast	257 (12.2%)	1 (5.9%)	258 (12.1%)	
Midwest	196 (9.3%)	3 (17.6%)	199 (9.4%)	
West	146 (6.9%)	5 (29.4%)	151 (7.1%)	
Unknown	24 (1.1%)	0 (0.0%)	24 (1.1%)	
Comorbidities				
Neoplasm	531 (25.2%)	4 (23.5%)	535 (25.2%)	0.874
Type 2 diabetes mellitus	237 (11.2%)	6 (35.3%)	243 (11.4%)	0.002
COPD	164 (7.8%)	4 (23.5%)	168 (7.9%)	0.017
Chronic kidney disease	161 (7.6%)	5 (29.4%)	166 (7.8%)	< 0.001
Heart failure	125 (5.9%)	2 (11.8%)	127 (6.0%)	0.312
Inflammatory bowel disease	113 (5.4%)	3 (17.6%)	116 (5.5%)	0.026
Systemic connective tissue disease	53 (2.5%)	1 (5.9%)	54 (2.5%)	0.38
Transplant	50 (2.4%)	1 (5.9%)	51 (2.4%)	0.347
Liver disease	43 (2.0%)	3 (17.6%)	46 (2.2%)	< 0.001
Pulmonary fibrosis	30 (1.4%)	2 (11.8%)	32 (1.5%)	< 0.001
Aplastic anaemia	27 (1.3%)	1 (5.9%)	28 (1.3%)	0.098
Type of sporotrichosis				
Lymphocutaneous	199 (9.4%)	1 (5.9%)	200 (9.4%)	0.616
Pulmonary	57 (2.7%)	2 (11.8%)	59 (2.8%)	0.024
Osteoarticular	22 (1.0%)	0 (0.0%)	22 (1.0%)	0.672
Disseminated	21 (1.0%)	2 (11.8%)	23 (1.1%)	< 0.001
Cerebral	2 (0.1%)	0 (0.0%)	2 (0.1%)	0.899
Antifungal therapy				
Itraconazole	475 (51.5%)	8 (66.7%)	483 (51.7%)	0.297
Fluconazole	70 (7.6%)	0 (0.0%)	70 (7.5%)	0.321
Posaconazole	17 (1.8%)	0 (0.0%)	17 (1.8%)	0.635
Voriconazole	12 (1.3%)	0 (0.0%)	12 (1.3%)	0.691
Isavuconazole	4 (0.4%)	0 (0.0%)	4 (0.4%)	0.819
Amphotericin B	18 (2.0%)	0 (0.0%)	18 (1.9%)	0.625
Echinocandin	7 (0.8%)	0 (0.0%)	7 (0.7%)	0.762
Laboratory values, mean (SD)				
WBC (×10^3^ cells/μL)	8.4 (4.5)	7.7 (3.4)	8.4 (4.5)	0.752
CD4 (cells/μL)	576.0 (.)	65.5 (62.9)	235.7 (298.1)	0.095
Haemoglobin (g/dL)	13.1 (1.9)	13.7 (2.2)	13.1 (1.9)	0.502
Haematocrit (%)	39.6 (5.4)	29.4 (20.6)	39.4 (6.1)	< 0.001
Platelets (×10^3^ cells/μL)	250.7 (93.6)	177.0 (48.8)	249.3 (93.4)	0.118
ALT (U/L)	30.8 (35.7)	24.2 (7.8)	30.7 (35.4)	0.716
AST (U/L)	28.6 (26.9)	29.7 (10.1)	28.6 (26.7)	0.945
Creatinine (mg/dL)	1.0 (0.8)	1.0 (0.3)	1.0 (0.8)	0.97
CRP (mg/L)	34.4 (61.2)	8.0 (.)	34.1 (60.8)	0.669
LDH (U/L)	239.3 (244.5)	160.0 (.)	234.4 (237.0)	0.758
Outcomes				
Hospitalisation	14 (0.8%)	0 (0.0%)	14 (0.8%)	0.75
ICU admission	16 (0.9%)	0 (0.0%)	16 (0.9%)	0.733
1‐year mortality	358 (17%)	3 (17.6%)	361 (17%)	0.943

Abbreviations: CD4, cluster of differentiation 4; COPD, chronic obstructive pulmonary disease; CRP, C‐reactive protein; HIV, human immunodeficiency virus; ICU, intensive care unit; LDH, lactate dehydrogenase; WBC, white blood cell count.

Pulmonary (11.8% vs. 2.7%, *p* = 0.024) and disseminated (11.8% vs. 1.0%, *p* < 0.001) disease were more common among individuals with HIV, whereas lymphocutaneous sporotrichosis predominated among those without HIV (9.4% vs. 5.9%, *p* = 0.616). Antifungal treatment patterns were comparable between groups. Itraconazole was the most frequently prescribed agent among people with HIV (66.7%) and those without HIV (51.5%) (*p* = 0.30), while amphotericin B and other azoles were infrequently used. Clinical outcomes, including one‐year mortality (17.6% vs. 17.0%, *p* = 0.94), hospitalisation (0% vs. 0.8%) and ICU admission (0% vs. 0.9%), did not differ by HIV status.

### 3.4 Lymphocutaneous Sporotrichosis Subgroup

Among the 200 cases of lymphocutaneous sporotrichosis, nearly all patients were HIV‐negative (99.5%). Common comorbidities included neoplastic disease (13%), diabetes mellitus (9.5%), heart failure (8%) and COPD (8%). Inflammatory markers were elevated (mean CRP of 49.6 mg/L and ferritin of 2441 ng/mL), and one‐year mortality was 22.5%. The most commonly used antifungal was itraconazole (55%).

## 4 Discussion

In this global real‐world cohort, sporotrichosis predominantly affected middle‐aged adults with chronic comorbidities, most notably malignancy and type 2 diabetes mellitus, and was associated with a one‐year mortality rate of 17%. These findings are consistent with previously reported overall mortality estimates of 10%–22% [13] and with mortality rates of 37%–42% reported for disseminated disease [3, 8]. Outcomes appear to be strongly influenced by host immune status, the magnitude of the inflammatory response and the promptness of antifungal therapy [8, 10]. Despite its global distribution and clinical relevance, sporotrichosis remains substantially underrecognised, in part because it is not a reportable disease in most countries [11]. Consequently, population‐level epidemiologic data are fragmented, and the burden of disease outside endemic regions remains poorly defined.

Non‐survivors in this study were more likely to have lymphocutaneous or disseminated disease, underlying malignancy, and markedly elevated ferritin concentrations. These findings illustrate the interplay between impaired host immunity, extensive tissue injury and fungal virulence in determining disease severity. Although traumatic inoculation remains the classical route of infection, alternative pathways, such as inhalation of conidia and zoonotic transmission, have become increasingly important [14]. The host immune response appears to be the principal determinant of clinical phenotype: immunocompetent individuals generally develop localised cutaneous or lymphocutaneous lesions, whereas immunocompromised hosts are predisposed to disseminated disease.

Approximately one‐quarter of the cohort had a neoplastic disease. However, hematologic malignancies accounted for only 4.6%, suggesting that profound or treatment‐induced immunosuppression was relatively uncommon and may account for the predominance of lymphocutaneous rather than disseminated disease. Few patients had autoimmune diseases or HIV. Among those with HIV, the mean CD4 count was 65 cells/μL, lower than the mean of 104 cells/μL reported in a systematic review of 37 coinfected patients [3]. One‐year mortality did not differ by HIV status, a finding likely attributable to the small number of HIV‐positive individuals, limited representation of untreated or advanced HIV disease and incomplete antiretroviral therapy or virologic data within TriNetX.

Marked hyperferritinemia observed among non‐survivors suggests that macrophage activation and associated systemic inflammation may contribute to adverse outcomes [15]. Although ferritin is not thought to play a direct pathogenic role in sporotrichosis, it plausibly functions as a surrogate marker of immune dysregulation and cytolysis, as described in other invasive fungal infections [15]. Most cases of sporotrichosis remain localised and do not elicit the severe hyperinflammatory states observed in hemophagocytic lymphohistiocytosis or severe sepsis [16]. Nevertheless, the association between elevated ferritin and mortality observed in this cohort warrants further investigation to evaluate ferritin's utility as a biomarker of disease severity and immune activation.

Itraconazole was the most commonly prescribed antifungal, in accordance with the Infectious Diseases Society of America (IDSA) guidelines, which recommend itraconazole for the treatment of cutaneous and lymphocutaneous disease [3, 13, 17, 18]. Amphotericin B, the recommended induction agent for disseminated or severe infection, was rarely used (< 2%), which may reflect underrecognition of severe disease, incomplete capture of inpatient data or deviation from guideline‐concordant therapy. The occasional use of fluconazole, posaconazole, voriconazole, isavuconazole and echinocandins, agents with variable, limited or unknown activity against *Sporothrix* species [11, 19, 20, 21], likely reflects empiric therapy before pathogen identification or therapeutic uncertainty. These treatment patterns highlight the need for heightened clinical awareness, enhanced diagnostic capabilities, and stricter adherence to standardised treatment protocols.

The geographic distribution observed in this cohort mirrors known ecological determinants that favour the proliferation of *Sporothrix* species [11]. Sporotrichosis is most common in tropical and subtropical climates across multiple continents. Within the United States, cases clustered in southern states, where higher humidity and temperature promote fungal persistence in soil and vegetation [22, 23, 24]. Occupational exposures, particularly in outdoor and agricultural settings, likely increase the risk of infection, while socioeconomic disparities may further influence exposure likelihood and access to timely diagnosis. Although zoonotic transmission is well documented in South America [24], the absence of exposure‐level variables in this dataset precluded assessing their contribution to observed infection patterns. These findings reinforce the importance of ongoing public health surveillance and environmental studies to better understand the ecological and socioeconomic factors driving sporotrichosis.

This study has significant limitations inherent to retrospective analyses of administrative and EHR‐based data. Case identification relied on ICD coding, which may lead to misclassification of sporotrichosis despite internal validation. We were unable to obtain additional geographic information for those patients outside the United States. Microbiologic and histopathologic data required for a definitive diagnosis per EORTC/MSGERC criteria were unavailable, precluding species‐level analysis. Some patients had diagnoses made decades ago, which may have contributed to the omission of observations for key variables, such as race/ethnicity. Laboratory completeness varied, and antifungal dosing, treatment duration, and adherence could not be evaluated. Mortality data were limited to available death records, and causes of death could not be verified. Mortality ascertainment was limited to linked records and may underestimate deaths occurring outside contributing systems. Finally, small subgroup sizes, particularly among patients with HIV, limit the precision of comparative analyses.

Despite these constraints, this study represents one of the largest real‐world evaluations of sporotrichosis, encompassing diverse healthcare systems and capturing longitudinal treatment and outcome data across multiple continents.

In this multinational cohort of over 2000 adults, sporotrichosis was associated with a one‐year mortality of 17%. Poor outcomes were observed in older patients and those with malignancy, lymphocutaneous or disseminated disease and systemic inflammation characterised by hyperferritinemia. Itraconazole was the predominant antifungal agent; however, the infrequent use of amphotericin B for severe infections and the use of agents with limited or unknown activity against sporotrichosis indicate potential gaps in recognition and guideline‐concordant management. These findings underscore the global clinical relevance of sporotrichosis, extending beyond traditional endemic regions. Earlier diagnosis, optimisation of antifungal therapy, and the incorporation of inflammatory markers into risk stratification may improve patient outcomes and inform future prevention and surveillance strategies.

## Author Contributions

**Samantha Schapiro:** data curation, formal analysis, investigation, methodology, project administration, resources, validation, writing – original draft. **Nicholas Pulciano:** data curation, methodology, validation, writing – original draft. **Julian Galindo‐Ramirez:** data curation, methodology, validation, writing – original draft. **Nicholas Rau:** writing – review and editing. **Jose Tuells:** resources, supervision, writing – review and editing. **Nelson Iván Agudelo Higuita:** supervision, visualization, writing – review and editing. **George R. Thompson III:** conceptualization, supervision, writing – review and editing. **Daniel B. Chastain:** data curation, supervision, visualization, writing – original draft, writing – review and editing. **Andrés F. Henao‐Martínez:** conceptualization, investigation, writing – original draft, methodology, validation, visualization, writing – review and editing, software, formal analysis, project administration, data curation, supervision, resources.

## Conflicts of Interest

G.R.T. reports grant support and consulting fees from Astellas, Basilea, Cidara, GSK, F2G, Melinta and Mundipharma. A.F.H.‐M. received educational funds from F2G Novel Therapeutics and is a Co‐PI on the MARIO trial, Investigating Ibrexafungerp as a non‐azole, oral step‐down for patients with invasive candidiasis and candidemia. All other authors report no potential conflicts of interest.

## Supporting information


**Table S1:** Identification of sporotrichosis cases by ICD‐9‐CM and ICD‐10‐CM codes.
**Table S2:** ICD‐10‐CM codes for key comorbidities included in the analysis.
**Table S3:** Logical observation identifiers, names and codes (LOINC) for laboratory parameters.
**Table S4:** RxNorm codes for antifungal medications.
**Table S5:** Outcome definitions by CPT, HL7 and ICD‐10‐CM codes.
**Table S6:** Multivariable Cox Regression analysis for one‐year mortality.

## Data Availability

The data that support the findings of this study are available on request from the corresponding author. The data are not publicly available due to privacy or ethical restrictions.
