# Supplementary material for: Epidemiology and Outcomes of Sporotrichosis: A Descriptive Real‐World Analysis From a Global Cohort
Source: Mycoses. 2026 Feb 24;69(2):e70152. doi: 10.1111/myc.70152 (PMC12930329; doi:10.1111/myc.70152)
Supplement: Supplementary file 1 — Table S1: Identification of sporotrichosis cases by ICD‐9‐CM and ICD‐10‐CM codes. Table S2: ICD‐10‐CM codes for key comorbidities included in the analysis. Table S3: Logical observation identifiers, names and codes (LOINC) for laboratory parameters. Table S4: RxNorm codes for antifungal medications. Table S5: Outcome definitions by CPT, HL7 and ICD‐10‐CM codes. Table S6: Multivariable Cox Regression analysis for one‐year mortality. [file MYC-69-e70152-s001.docx]

**Supplementary Online Content**

**Table S1. Identification of Sporotrichosis Cases by ICD-9-CM and ICD-10-CM Codes**

**Table S2. ICD-10-CM Codes for Key Comorbidities Included in the Analysis**

**Table S3. Logical Observation Identifiers, Names, and Codes (LOINC) for Laboratory Parameters**

**Table S4. RxNorm Codes for Antifungal Medications**

**Table S5. Outcome Definitions by CPT, HL7, and ICD-10-CM Codes**

**Table S6. Multivariable Cox Regression analysis for one-year mortality.**

**Table S1. Identification of sporotrichosis cases by International Classification of Diseases, Ninth Revision, (ICD-9) or International Classification of Diseases, Tenth Revision, Clinical Modification (ICD-10-CM) diagnosis codes.**

| **Code** | **Description** |
| --- | --- |
| B42, 117.1 | Sporotrichosis |

**Table S2. International Classification of Diseases, Tenth Revision, Clinical Modification (ICD-10-CM) diagnosis codes for underlying comorbidities.**

| **Code** | **Description** |
| --- | --- |
| B20 | HIV infection |
| C00-D49 | Neoplasms |
| D70-D77 | Blood disorders |
| D60-D64 | Aplastic anemias and bone marrow failure syndromes |
| D61 | Aplastic Anemia |
| E11 | Diabetes mellitus |
| J84.10 | Pulmonary Fibrosis |
| K74 | Hepatic fibrosis and cirrhosis |
| M30-M36 | Systemic connective tissue disorders |
| N18 | Chronic kidney disease |
| Z94 | Transplanted organs or tissues |
| I50 | Heart failure |

**Supplementary Table S3. Logical Observation Identifiers, Names, and Codes (LOINC) for laboratory values**

| Code | Description |
| --- | --- |
| 9015 | White blood cell count |
| 24467 | CD4 cells |
| 9048 | Aspartate aminotransferase |
| 9044 | Alanine aminotransferase |
| 9024 | Serum creatinine |
| 9042 | Ferritin |
| 9063 | C-reactive protein |
| 9052 | Lactate dehydrogenase |
| 718 | Hemoglobin |
| 4544 | Hematocrit |
| 30341 | Erythrocyte Sedimentation Rate |
| 75241 | Procalcitonin |
| 777 | Platelets |

**Supplementary Table S4**. RxNorm codes for medications

| Code | Description |
| --- | --- |
| 28031 | Itraconazole |
| 4450 | Fluconazole |
| 8640 | Posaconazole |
| 121243 | Voriconazole |
| 1608322 | Isavuconazole |
| 732, 42527 | Amphotericin B |
| 140108, 341018, 325887 | Echinocandin |

**Supplementary Table S5**. Outcomes based on Current Procedural Terminology (CPT) codes, HL7 Terminology, and International Classification of Diseases, Tenth Revision, Clinical Modification (ICD-10-CM) diagnosis codes

| Code(s) | Description |
| --- | --- |
| 1013659, 1013660, 1013699  1013729, 1014309, 99291 | Hospitalization |
| 1013729, 1014309, 99291 | Intensive Care Unit services |

**Table S6. Multivariable Cox Regression analysis for one-year mortality.**

| **Variable** | **Hazard Ratio** | **95% Confidence Interval** | **p-value** |
| --- | --- | --- | --- |
| Age (per year) | 1.01 | 0.99 – 1.03 | 0.470 |
| Male gender (vs. female) | 1.68 | 0.84 – 3.35 | 0.144 |
| Ethnicity (ref: Hispanic) |  |  |  |
| Not Hispanic | 0.82 | 0.31 – 2.17 | 0.697 |
| Region | 1.21 | 0.95 – 1.54 | 0.125 |
| Neoplasm | 0.97 | 0.45 – 2.07 | 0.931 |
| HIV infection | 0.32 | 0.03 – 4.22 | 0.390 |
| Disseminated sporotrichosis | 3.72 | 0.82 – 16.96 | 0.090 |
| Hematocrit (per 1% increase) | 0.94 | 0.89 – 0.99 | **0.012** |
